# *Khaya senegalensis* inhibits piroxicam mediated gastro-toxicity in wistar rats

**Published:** 2014

**Authors:** Fatima Nnawodu Ishaq, Abdulkadir Umar Zezi, Temidayo Olutoyin Olurishe

**Affiliations:** *Department of Pharmacology and Therapeutics, Ahmadu Bello University, P M B 1045, Zaria-Nigeria*

**Keywords:** *Drug interaction*, *Gastro-toxicity*, *Khaya senegalensis*, *Piroxicam*

## Abstract

**Objective**: The purpose of this study was to investigate the effects of piroxicam co-administration with ethanolic stem-bark extract of *Khaya senegalensis *on biomarkers of oxidative stress and gastro-toxicity in Wistar rats.

**Materials and Methods**: Thirty healthy male and female Albino Wistar rats (190-220 g) were grouped into six (n = 5) with designated treatments including: Normal saline, piroxicam (20 mg/kg), extract (200 and 400 mg/kg) alone and both doses of the extract co-administered with piroxicam. The drugs were administered orally to all the rats for fourteen consecutive days and on the fifteenth day, they were euthanized with chloroform inhalation. Blood samples and the stomachs were isolated for evaluation of the oxidative stress biomarkers and gastro integrity, respectively.

**Results**: The results of the study revealed that the levels of oxidative stress markers didn’t differ significantly between the groups receiving the extract alone, the extract in combination or piroxicam alone. Gross and histological observations of the stomach showed gastric mucosal changes and mild atrophic lesions in the piroxicam group only.

**Conclusion**: This study illustrates the interaction of *Khaya senegalensis *and piroxicam results in the gastro-protective beneficial effects. The extract’s outcome on various prostaglandin levels and synthesis is being considered towards possible elucidation regarding the exact mechanism of cytoprotection.

## Introduction

Since modern medicine stands quite close to traditional practices, it does not seem odd to witness an increase in the awareness and the general acceptability regarding the use of herbal drugs with orthodox preparations (Fasinu et al., 2010; Osemene et al., 2011). Many patients are unlikely to consider the risks of herbal self-medication and its association with drug interactions; therefore such cases are not usually reported (Wong and Townley, 2011). However, adverse reactions have been reported in herbal medicines when used concurrently with conventional or orthodox medicines (Oreagba et al., 2011 ▶). One of the most highly utilized classes of pharmaceutical agents in medicine are the Non-steroidal anti-inflammatory drugs (NSAIDs) (Simmons et al., 2004; Taubert, 2008 ▶). NSAIDs are known for their interaction with herbs, which are commonly used for pain and inflammation. Herbs may exacerbate some of the adverse effects of NSAIDs including gastrointestinal toxicity (Holden et al., 2004). *Khaya senegalensis *Desr. A. Juss. (Meliaceae) known by the English as African mahogany; Yoruba as ‘oganwo’; Hausa as ‘madachi’, and known as ‘ono’ in Igbo (Ibrahim et al., 2006 ▶) has been established to possess analgesic and anti-inflammatory effects (Lompo et al., 2007 ▶), anti-diarrhoeal effects (Nwosu et al., 2012 ▶), antioxidant effects (Karou et al., 2005; ▶ Lompo et al., 2007 ▶), hypoglyceamic effects (Etuk and Mohammed, 2009 ▶; Sollu et al., 2010 ▶; Kolawale et al., 2012 ▶) ulcer protective properties (Nwafor et al., 1996 ▶), and anti-malarial effects (Nwosu et al., 2012 ▶). People taking NSAIDs may also use *Khaya senegalensis *to further relief their pain or to benefit from its other medicinal properties. Hence, the efficacy and safety of the co-administered drugs may not be objectively isolated and assessed. The study was aimed to evaluate the effect of the concurrent administration of ethanolic stem-bark extract of *Khaya senegalensis *and piroxicam on some markers of oxidative stress and gastro-toxicity.

## Materials and Methods

**Plant collection, authentication and extraction**

The stem bark of *Khaya senegalensis* was collected in November, 2012 in Zaria Nigeria and the plant authentication was done by Umar Gallah at the Herbarium unit of the Department of Biological Sciences, Ahmadu Bello University, Zaria-Nigeria, where a voucher specimen number (900181) was assigned by comparing with the deposited specimen available as reference. The collected stem bark was air dried to constant weight and pulverized into powdered form. The powdered stem bark (900 g) was cold macerated, using 70% ethanol in distilled water over forty-eight hours. The filtrate was evaporated to dryness over a steam bath at 45°C, and resulted in a yield of 18.56 %.

**Materials, Drugs and chemicals**

These included whatman’s filter paper, board, Piroxicam (Hovid BHD, Malaysia), 96 % absolute alcohol (Sigma, USA), and Chloroform (Sigma, USA). All dilutions and drug preparations were done using distilled water. The extract was also prepared freshly through using distilled water before each experiment.

**Experimental animals**

Wistar rats of either sex, weighing 190-220 g, were used in the studies. The rats were purchased from the Animal House of the Faculty of Pharmaceutical Sciences, Ahmadu Bello University, in Zaria. They were maintained on standard rodent feed and water *ad libitum, *except when fasting was required. Rats were treated in accordance with the ‘Principles of Laboratory Animal Care’ (NIH publication no. 18-23, Revised, 1985). 

**Experimental design**

Animals were divided into six group (n = 5). The first group served as a physiological control receiving normal saline (10 ml/kg), while the second group received 20 mg/kg piroxicam as the negative control. The third and fourth groups received *Khaya senegalensis* extract at 200 and 400 mg/kg, respectively. The fifth and sixth groups received similar treatments as the third and fourth, but received 20 mg/kg of piroxicam concurrently in addition with the extract. These doses were selected based on the acute toxicity and antinocieptive studies that were previously carried out (Olurishe et al., 2013 ▶). All drug and extract administrations were done orally with the aid of a gavage needle after an overnight fasting. 

**Blood sampling**

On day 15, after euthanizing the animals with chloroform inhalation, blood samples were collected from the jugular vein of each rat into anti-coagulant free containers, each contained 2 ml (Eseyin et al., 2006 ▶). Blood samples were centrifuged at 3000 rpm, using a centrifuge (Centurion model GP (CD295-30) to get a clear serum. Serum determination of glutathione peroxidase (GP_X_) and malondialdehyde (MDA) were performed by using an automated analyzer, Audicom (AC 9900, USA). 

**Gross gastro-integrity assessment**

Gastro-toxicity observations like diarrhea, discolored stool, and abdominal constrictions were observed daily, during the 14 days administration of drug. Gross gastric lesion was observed on day 15 in all the test animals following euthanization. Gastric mucosal observations were done as described by (Magaji et al., 2007 ▶). The stomachs were excised and opened along the greater curvature. They were slowly washed with normal saline and then stretched out as much as possible on a Whatman’s filter paper pinned on a board. The ulcerated surface in each stomach was measured with a transparent millimeter scale rule and the result for each group was expressed as mean ulcer ± SEM (Standard Error of Mean). The stomach tissues were then preserved in 10 % formalin for histological observations.

**Histological examination**

The preserved stomachs (used for gross gastric assessment) were embedded in paraffin wax. They were then cut according to standard micro techniques onto glass slide and stained with hematoxylin and eosin (Sidahmed et al., 2013 ▶). Photomicrographs were carried out and the slides were examined microscopically by a histopathologist.

**Statistical analysis**

The results for quantitative/continuous variables were expressed as Mean ± standard error of mean. Data was analyzed with one way ANOVA that was followed by Tukey post hoc test, using version 20 of SPSS software while p values less than or equal to 0.05 were considered statistically significant.

## Results

**Effect of Two Weeks Co-administration of Piroxicam and Ethanolic Stem-Bark Extract on Glutathione peroxidase (GPx) and Malonedialdehyde** **(MDA)** **in Rats**

The results indicated that the levels of oxidative stress biomarkers, glutathione peroxidase (GPx) and malonedialdehyde (MDA) of rats exposed to 14 days treatment of the two doses (200 and 400 mg/kg) of the *Khaya senegalensis* extract, and concurrent piroxicam and the two doses of the extract on GPx and MDA levels were not significantly different as compared to normal saline and piroxicam groups (Table 1).

**Effect of two weeks co-administration of piroxicam and ethanolic stem-bark extract on gross gastro-integrity in rats**

In this study, only Piroxicam treated rats demonstrated mild diarrhea and abdominal constriction along with the whole body stretching that started on tenth day and continued till the end of the study. Mucosa lesions including perforations were observed in piroxicam treated group alone, and the ulcer index in this group was found to be 45.63 ± 12.55 (mm).

**Table 1:** Effect of two weeks co-administration of piroxicam and the ethanolic stem-bark extract on glutathione peroxidase (GPx) and malonedialdehyde (MDA) of piroxicam administration in rats

**Group**	**MDA (µg/ml)**	**GP**_X _ **(IU/L)**
NS	1.62±0.09	43.20±4.31
PC	1.60±0.13	38.58±4.52
E200	1.42±0.07	34.40±2.23
E400	1.94±0.17	52.4±3.75
PC+E200	1.66±0.23	38.40±1.89
PC+E400	1.46±0.16	37.80±3.18

Data shown is Mean ± SEM, no significant difference when compared to normal saline and piroxicam.. (One-way ANOVA followed by Tukey’s post hoc test). NS: normal saline, PC: piroxicam 20 mg/kg, E_200_: extract 200 mg/kg, E_400_: extract 400mg/kg, MDA: malonedialdehyde, GPx: glutathione peroxidase

**Table 2:** Effect of two weeks co-administration of piroxicam and ethanolic stem-bark extract on the gross gastro integrity of piroxicam administration in rats

**Group**			**Gross Observation**	**Ulcer index (mm )**			
NS		UMC			-		
PC	MLP				45.63 ± 12.55		
E_200_		UMC			-		
E_400_		UMC			-		
PC + E_200_		UMC			-		
PC +E_400_		UMC			-		

Data is shown as Mean ± SEM, n=5, - = nil, NS: normal saline, PC: piroxicam 20 mg/kg, E_200_: ethanolic stem-bark extract 200 mg/kg, E_400_: ethanolic stem-bark extracts 400 mg/kg, UMC: unremarkable mucosal changes, MLP: mucosal lesion and perforations.

**Table 3:** Effect of two weeks co-administration of piroxicam and ethanolic stem-bark extract on gastric histology in rats

**Group**	**Histological observations**
NS	UMC
PC	MAC
E_200_	UMC
E_400_	UMC
PC + E_200_	UMC
PC +E_400_	UMC

Data is shown as NS: normal saline, PC: piroxicam 20 mg/kg, E_200_: ethanolic stem-bark extract 200 mg/kg, E_400_: ethanolic stem-bark extracts 400 mg/kg, UMC: unremarkable mucosal changes, MAC: mild atrophic changes.

**Effect of two weeks co-administration of piroxicam and ethanolic stem-bark extract on gastric histology in rats**

Only piroxicam treated group showed histo-architectural distortion (Table 3 and Figure II). No lesions were observed in the other treated groups (Table 3 and Figure I). 

**Figure 1:**
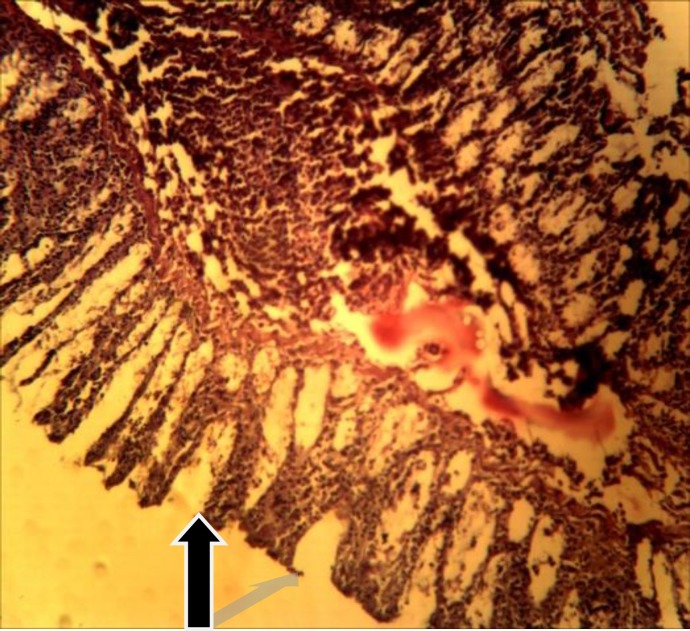
A representative stomach section showing unremakable mucosal changes with normal epithelia lining (H and E x 250).

**Figure 2:**
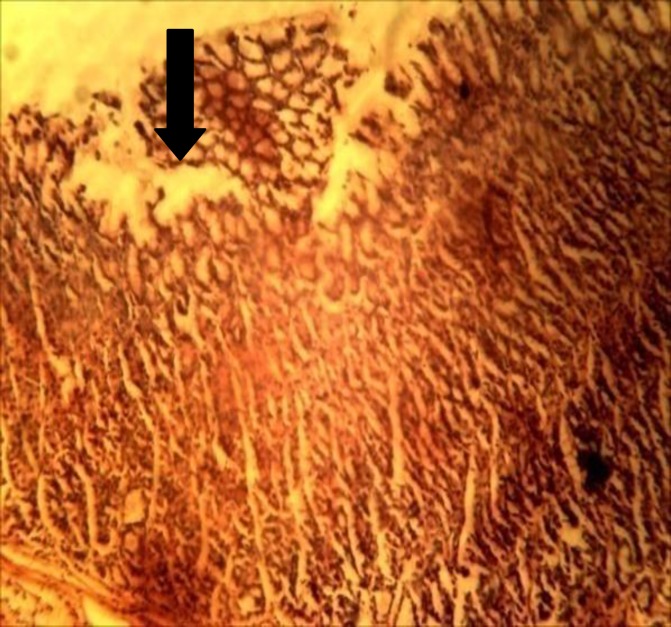
Stomach section of piroxicam treated group. Arrow showing gastric mucosa with

mild atrophic changes (MAC) (H and E x 250).

## Discussion

Glutathione peroxidase (GPx) levels of all treatment groups were similar, suggesting that the antioxidant activity of this enzyme was not altered by the combination therapy. The antioxidant enzyme defense system is made up of free radical scavengers including glutathione

peroxidase (GPX). Oxidative stress occurs when there is an over production of reactive oxygen species (ROS) and a decrease in antioxidant defenses (Selvakumar et al., 2013 ▶). Malonedialdehyde (MDA) level, also a biomarker for oxidative stress (an ending product of lipid peroxidation), was reduced in the group that received concurrent piroxicam and 400 mg/kg of the extract. Decreasing lipid peroxidation by this combination may be attributed to the antioxidant activity of Khaya senegalensis since it contains many phenolic compounds (Olurishe et al., 2013 ▶), which have inhibitory effects on lipid peroxidation. The offered extract protection of cells against oxidative stress may be due to scavenging free radicals. 

Gross gastro-toxicity and changes in histology of the stomach were observed only in the piroxicam treated group, representing an expected typical gastro toxicity from piroxicam. This impairment in mucosal defense is consequent to the suppression of prostaglandin synthesis and direct cell necrotic action of piroxicam, which led to the gastric ulceration and perforations. The gastrointestinal disorder that was due to 

sub-acute piroxicam toxicity has been reported earlier (Udegbunam et al., 2012 ▶). The stomach of animals that received the extract in addition to piroxicam did not show any gross mucosal lesions or histological alterations. This suggests the possibility of some direct protective effects of *Khaya senegalensis* in maintaining the gastric integrity of rats that are exposed to piroxicam. The gastrointestinal tract (GIT) is considered the major site for NSAID toxicity in humans and animals (Sale et al., 2005 ▶; Salawu et al., 2009 ▶). Piroxicam induces GIT damage by directly distorting the mucosa lining or through the inhibition of cyclooxygenase, thereby diminishing the production of prostaglandins and consequently lowering the mucosa defense (Kalra et al., 2011). Prostaglandins play a central role in protecting the gastric mucosa by stimulating the production of mucus, bicarbonate, surface hydrophobicity, mucosal blood flow, inhibition of hydrochloric acid secretion, and possible endothelial and epithelial cellular protection (Choquet et al., 1993; Jackson et al., 2000 ▶). Several reports have suggested that COX-1 protein and mRNA predominate in gastric tissue, which may account for the most endogenous prostaglandin synthesis and thus maintain the GIT mucosal integrity (McCormick et al., 2010).

*Khaya senegalensis* bark extract has been previously hypothesized to contain inhibitors of the cyclooxygenase-2 (COX-2) gene (Lompo et al., 2007 ▶). Selective COX-2 inhibitors caused a reduction in prostaglandin levels and oedema in areas of inflammatory lesions but had no effect on either gastric mucosal prostaglandin levels or integrity; therefore COX-2 inhibition will spare COX-1-induced cytoprotection of the gastrointestinal tract (Fosslien, 2000 ▶). Also, the ethanolic crude extract of the stem bark of *Khaya senegalensis* has been reported to possess free radical scavenging activities in protecting gastric mucosal damage (Kolawole et al., 2012 ▶). Nwafor et al., (1996) ▶ reported the anti-cholinergic, anti-histarminergic and anti-serotonergic properties of *Khaya senegalensis, proposing the* ability to reduce gastric acidity and trigger changes in ionic permeability in gastric mucosa.

 Phytochemical studies that we conducted earlier, revealed the presence of anthraquinones carbohydrates, flavonoids, glycosides, saponins, sterioids, tannins, and triterpenes (Olurishe et al., 2013 ▶). Some of these phytochemical constituents such as flavonoids, tannins, terpenoids, and saponin have been reported to have possible gastro protective effects (Salawu et al., 2009 ▶; Gadekar et al., 2010; Arumugam et al., 2011 ▶; Inas et al., 2011; Balamurugan et al., 2013 ▶). 

The findings from this study indicate that the ethanolic stem bark extract of *Khaya senegalensis* ameliorates piroxicam associated gastro-toxicity which could be via the enhancement of gastric mucosa protection and anti-oxidant effects. The concurrent use of *Khaya senegalensis* and piroxicam that has been previously displayed to enhance the antinociceptive actions of the latter yet offers further beneficial properties and requires detailed mechanistic studies.
